# Vibrotactile Stimulation Encoded by Beta and Gamma Bands Varies with Locations on the Upper Limbs

**DOI:** 10.3390/bioengineering13070793

**Published:** 2026-07-10

**Authors:** Sage R. N. Gatewood, Ajay T. Arul, Annalise X. Le, Ashif A. N. Zeesan, Yan Gai

**Affiliations:** Biomedical Engineering, School of Science and Engineering, Saint Louis University, 3507 Lindell Blvd, St. Louis, MO 63103, USA; sagegetsemails@gmail.com (S.R.N.G.); annalebmw@gmail.com (A.X.L.); zeesan.zeesan@slu.edu (A.A.N.Z.)

**Keywords:** EEG, somatosensory, tactile, location perception, gamma

## Abstract

Background: The perception of tactile locations is an important function of human’s somatosensory system during body movements and its interactions with the surroundings. Our previous study on the perception of locations found that the gamma-frequency band provides better decoding accuracy than all the lower frequencies on the legs. In the present study, we recorded electroencephalography (EEG) responses evoked by four vibrotactile stimulators placed on the arms of 18 human subjects. Methods: Human subjects were instructed to sit in a chair while somatosensory-evoked potentials were obtained using a 64-channel EEG. A linear classifier and an artificial neural network with 10 hidden-layer neurons were separately used to predict tactile locations based on EEG power obtained from various frequency bands. Results: We found that the beta (13–30 Hz) and high-gamma (50–100 Hz) bands can best predict the tactor locations on the arms. Interestingly, power information carried by the high-gamma band was uncorrelated to information contained in the lower frequency bands. Consequently, combining the beta and high-gamma bands significantly improved the prediction accuracy. Conclusions: Our findings prove that tactile location information can be decoded from EEG signals, which agrees with previous studies regarding the importance of the gamma band during tactile perception.

## 1. Introduction

Spatial localization of sensory input is a fundamental and even survival ability for human’s multiple sensory systems. Somatosensory and visual systems share certain common processing strategies in their peripheral systems [1], as mechanoreceptors and photoreceptors intrinsically encode the spatial locations of input stimuli. This contrasts with the auditory system, in which the inner hair cells are arranged according to the sound frequency. The identification of tactile-stimulation locations on the human body is a critical function for humans to interact safely and effectively with the environment. Information regarding a physical object’s shape, orientation, texture, temperature, and material properties may be inferred during tactile perception [2,3,4,5]. Studies have also demonstrated that tactile spatial perception plays a critical role in sensorimotor integration, body representation, and multisensory processing [6,7,8,9].

Previous investigations of tactile-location perception have primarily focused on psychophysical aspects, such as localization accuracy, spatial-location discrimination, and perceptual biases [10,11,12,13]. The brain’s ability to translate the location of a tactile input is sometimes referred to as tactile spatial remapping [8,14], especially when the remapping process is susceptible to various internal and external factors. For example, the location-discrimination acuity of vibrotactile perception across the arms is affected by spatial and temporal patterns of the vibrotactile stimuli [15]. Body postures, such as crossing the hands or arms, can clearly affect the spatial perception of tactile stimuli [1].

In rodents, neural decoding of tactile locations has been extensively studied by applying stimulation to different whiskers or digits, while recording neuronal activity from the primary somatosensory cortex [16,17,18]. Studies using high-density stimulations and optogenetic approaches have demonstrated that spatially distributed neuronal ensembles in rodents’ somatosensory cortex (i.e., the barrel cortex) encode tactile position and texture information with high temporal precision [19,20].

In humans, fewer studies have investigated whether tactile locations can be directly decoded using non-invasive neurophysiological techniques. In other words, previous studies mostly focused on detecting the occurrence of the tactile stimulation rather than the location. One study using functional magnetic resonance imaging (fMRI) reported that tactile distance judgments and contact judgments involve distinct cortical networks [21]. Meanwhile, studies on tactile remapping have examined brain responses to tactile input on arms and hands, often fingers, using electroencephalography (EEG) [1,22,23]. Those remapping studies focus on the distribution and propagation of early and late components of somatosensory-evoked potentials (SEPs) right after a tap has been applied. Because the tactile stimulation is typically brief, various peaks in the temporal waveforms of SEPs are analyzed. A major goal is to explore whether intrinsic or extrinsic spatiotopic encoding dominates tactile remapping.

Alternatively, steady-state tactile responses have been examined using vibrational stimulation and frequency analysis. He and Contreras-Vidal [24] applied vibrotactile stimulation to single or both hands of their human subjects. They chose to examine the delta band (0.4–4 Hz) with the rationale that “this band has been reported to contain motor-related information”. Wang, et al. [25] applied vibrotactile stimulation to four locations on the right arm of their human subjects and extracted the oscillatory power of EEG filtered from different frequency bands for the decoding the tactile location. Although they showed that the high-beta frequency band (25–32 Hz) yielded the highest decoding performance, they did not examine EEG signals above 32 Hz. Here, we would like to point out an interesting EEG study that examined late brain responses separately to *what* and *where* of the tactile input [26]. It showed that the *what* pathway is strongly lateralized, depending on the side of tactile stimulation, whereas the *where* pathway is more bilateral and does not differ with which hand. The finding indicates that the perception of tactile locations may rely on unique mechanisms.

Accumulating evidence suggests that gamma-band activity (>30 Hz, which may extend up to 200 Hz using more invasive recording approaches) carries important information regarding various tactile features, such as texture perception, vibration frequency, multisensory integration, and pain-related processing [27,28,29]. Using electrocorticography (ECoG), Ryun, et al. [27] showed that the high-gamma band (50–140 Hz) can encode very high vibration frequencies. In addition, gamma oscillations in the somatosensory cortex are associated with tactile attention, perceptual binding, movement-related sensory gating, and cortical communication during somatosensory processing. The EEG study on human walking [30] showed that gamma oscillations are reduced during the stance phase of walking. A magnetoencephalography (MEG) study on somatosensory gating [31] showed reduced coupling between gating and gamma oscillations during panic disorder. Another MEG study showed that gamma amplitude encodes tactile intensity, a feature related to pain perception [32]. A study in mice showed that fast-spiking interneurons, which are considered as the origin of the gamma frequency, can enhance the detection of week tactile stimuli [33].

In our previous human EEG study [34], four vibrotactile stimulators were attached to the lower body, particularly to tissue regions involved in sitting. We examined EEG power up to the low-gamma frequency (30–50 Hz) and found it to be the best frequency band in decoding tactile locations. We also examined the effect of different types of cushions over prolonged sitting in terms of sensitivity to vibrotactile locations. Female subjects generally showed less degradation in the sensitivity than male subjects. In the present study, vibrotactile stimulation was delivered to the epidermis of both arms while SEPs were recorded, in a way similar to the leg study. A major goal is to examine if the high-gamma band, which extends the signal frequency up to 100 Hz, contains useful information for decoding tactile locations on the arms. Specifically, EEG power was extracted from the conventional delta, theta, alpha, beta, low and gamma bands using 63 electrode channels to form a 63-feature input for each frequency band. Tactile locations were decoded by training a linear classifier and an artificial neural network (ANN) using the 63 power features. Classification performance was then evaluated under multiple experimental conditions, including the frequency band, tactile-stimulation intensity, and gender. An unexpected finding is that different tactile locations may be best decoded with different frequency bands. In particular, the high-gamma band contained non-redundant information that is uncorrelated to the low-frequency bands. Given this observation, we examined the possibility that combining multiple frequencies may yield even better classification results.

## 2. Materials and Methods

### 2.1. Experimental Setup

The experimental protocol was approved by the Institutional Review Board of Saint Louis University (Protocol #29002, “Electroencephalography, sound detection, eye-tracking, and somatosensory-evoked potentials towards applications in prosthetics”, approved on 26 February 2018). A written consent was waived and replaced by a recruitment statement because the study involved no more than minimal risk.

Vibrotactile stimulation waveforms were created using a Tactor Development Kit (TDK, Engineering Acoustics, Inc., Casselberry, FL, USA) implemented in MATLAB (MathWorks, Natick, MA, USA; R2022b). Each trial consisted of a 1 s, 25-Hz sinusoid, followed by 3 s of silence. Two vibration intensities were tested by adjusting the gain value in the TDK program. The “high-intensity condition” produced vigorous vibrations that could be clearly perceived. This was the single condition used in our previous study of tactile locations on the legs [34]. Here, we also added a “low-intensity condition” by setting the gain to half of the value used in the high-intensity condition.

**Figure 1:**
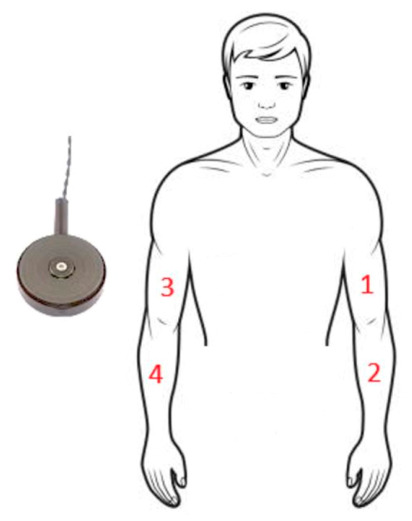
**Left**, a vibrational stimulator, “C-2 tactor”, with a diameter of 1¼ inches. **Right**, placement of the tactors, which were secured to the subject’s arms via cuff straps. The red numbers marked on the arms indicate the sequence of the stimulation.

Eighteen human subjects (aged 19–26), including nine women and nine men, participated in the study. The subjects had no history of neuromuscular disorders. After the subject sat down in a chair, a 2-D array of vibrators, called C-2 “tactors” (1¼-inch diameter, Figure 1, left), were secured to the subject’s arms with cuff straps. Figure 1 (right) shows the tactor placement on the arms. The two arms were naturally positioned at the body’s two sides when the subject was sitting in the chair. Vibrations of the tactors were controlled by an 8-channel EAI Universal Controller (Engineering Acoustics, Inc., Casselberry, FL, USA). During each recording session, 50 stimulations were applied consecutively at each tactor location, taking an overall duration of 13.4 min. Overall, 10 sessions of low-intensity and 10 sessions of high-intensity conditions were collected from each human subject. The room temperature ranged between 20 and 22 °C.

EEG signals were obtained with a 64-channel portable system (ANT Neuro) that comprised a head cap, an amplifier, and a Windows tablet computer. At the onset of each stimulation, the computer sent a trigger signal via a StimTracker (Cedrus, San Pedro, CA, USA) to mark the event in the EEG amplifier. CPz is the default reference during acquisition. For offline signal processing, the “common average montage” [35] was applied by subtracting the average from each of the 63 active electrodes. When there were environmental noise and other types of artifacts that were unrelated to brain signals, those unwanted signals should also exist in the common average and get subtracted out from the active electrodes. Even when there were residues, they contained no useful information related to tactile locations.

### 2.2. Signal Processing and Classification

Blinking and major movement artifacts were removed from EEG recordings during pre-processing by applying an Independent-Component-Analysis (ICA) approach [36] to the recorded signals. The ICA algorithm performs blind-source separation [37] to uncover independent signal sources recorded with multiple electrodes. The output channels may contain independent components that clearly show blinking or other artifacts. Removing those channels during the inverse linear transformation will not damage useful EEG information.

SEPs were bandpass-filtered between 0.5 and 100 Hz using a 200th-order finite-impulse-response filter. Given that each tactile stimulation lasted for 1 s and the response often lasted longer, we extracted the SEPs between 0 and 2 s. First, we will demonstrate that classification of the tactile locations based on the temporal waveforms of SEPs failed to generate significant results. Therefore, we resorted to the frequency-power measurement, which is more suitable for stead-state responses anyway. On each stimulation trial, the power of the 2-s SEP was computed as the normalized signal power, $\emptyset_{i}$ for each frequency band, i. Six frequency bands were defined according to previous studies [38,39], namely the delta (0.5–4 Hz), theta (4–7 Hz), alpha (8–12 Hz), beta (13–30 Hz), low gamma (30–50 Hz) and high gamma (50–100 Hz).

**Figure 2:**
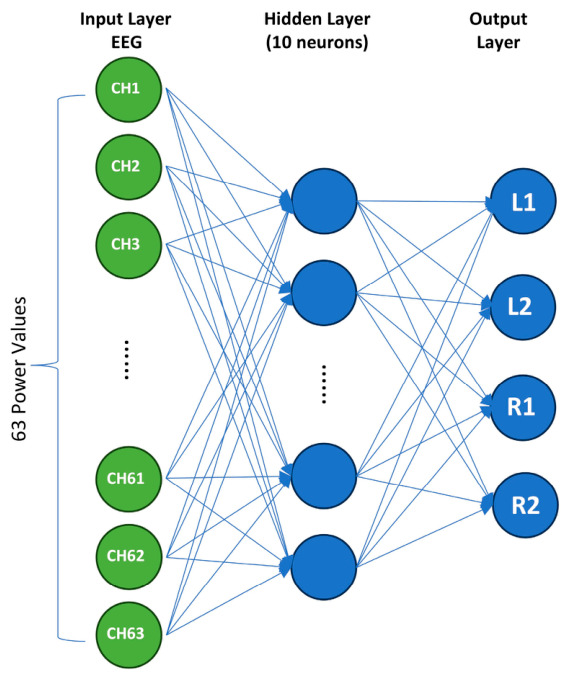
Structure of the ANN used for classifying the EEG signals. The input vector *x* in Equation (1) was fed to the input layer of the network.

Two classification algorithms were applied. For each frequency band, the power values of all 63 electrodes formed the input vector,

(1) $$x=[\emptyset_{1}\;\emptyset_{2}\;\cdots \;\emptyset_{63}].$$

x was then fed to a Linear-Discriminate-Analysis (LDA) classifier [40]. In the previous leg study [34], an ANN was adopted because it generated better results than the LDA. In the present arm study, a similar ANN was applied to the single-frequency data. Figure 2 shows the network structure. The number of input neurons was determined by the number of EEG channels (i.e., 63). Neurons in the output layer corresponded to the four tactile locations. On each trial, one of the four output neurons had a positive input, indicating the decision of the ANN classifier. There were 10 neurons in the hidden layer. The MATLAB function *fitnet()* was used to construct and train the ANN.

Both the LDA and ANN classifiers were evaluated with the same 10-fold cross-validation approach. For each human subject, 2000 data samples (four tactor locations, each having 500 trials) were randomly split into 10 groups. Each of the 10 groups then took turns being the test group on each run, while the other nine groups of data samples formed the training pool. In the end, a confusion matrix was constructed over the 10 runs. The classification accuracy, γ, was derived from the confusion matrix by normalizing the sum of the diagonal values with the sum of the entire confusion matrix (i.e., 2000).

To examine the significance of classification accuracy, the 99% confidence interval, CI, was computed using the Wilson score interval [41] as,

(2) $$CI=\frac{\gamma +\frac{z^{2}}{2N}\pm z\sqrt{\frac{\gamma (1-\gamma )}{N}+\frac{z^{2}}{4N^{2}}}}{1+\frac{z^{2}}{N}}.$$

Here, N is the number of data points (N=500·4=2000). z is 0.59 for 99% confidence. CI measures the degree of certainty in the sampling method. It is codetermined by the amount of variation and the total number of samples (i.e., N). If the lower boundary, γ−CI, is greater than chance level (i.e., 25% for four locations), a statistical significance is reached.

When comparing the accuracy values, $\gamma_{1}$ and $\gamma_{2}$, across two experimental conditions, the two-proportion *z*-score was computed to examine the statistical difference [42].

(3) $$z=(\gamma_{1}-\gamma_{2})/\sqrt{\frac{\gamma_{1}\left(1-\gamma_{1}\right)}{N}+\frac{\gamma_{2}\left(1-\gamma_{2}\right)}{N}}.$$

When multiple comparisons of the same γ were performed, the Bonferroni correction was applied to reduce the risk of false positive.

In addition, the input to the LDA classifier was a 63-feature power vector, including power values combined across 63 EEG electrodes. To examine which electrodes contributed most to the decisions, a one-way Analysis of Variance (ANOVA) test was applied to each electrode, and the *F* value served as an indicator of significance. A brain topography made of *F* values illustrates the electrode locations where the tactile locations mattered.

## 3. Results

We first examined classification performance using power values derived from single frequency bands. Since there was evidence showing that different frequencies may prefer different tactile locations, we then attempted to improve the classification accuracy by combining frequency bands. In the end, we examined if gender influenced the decoding of tactile locations on the arms, since we previously found an effect in the leg study [34].

### 3.1. SEPs and Power Topology

**Figure 3:**
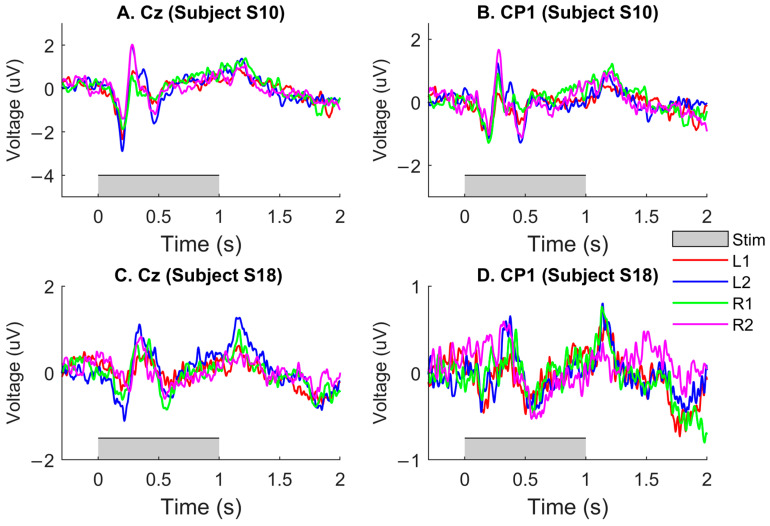
Selective SEPs obtained with Electrodes Cz and CP1 for two subjects. The stimulation intensity was low. The SEPs were raw and unfiltered. Different colors represent different tactor locations. Each trace is an average of 500 trials. The shaded area marks the duration of the tactile stimulation.

Figure 3 shows typical SEPs from two subjects obtained with two electrodes. The gray area indicates the duration of tactile stimulation. Traces with different colors are averages of 500 trials for each tactile location. Typical N1, P2, and N3 can be observed with most subjects with these two electrodes. Here, the traces were obtained with the low tactile-stimulation intensity. When the intensity increased, responses were very similar to Figure 3.

Although there can be consistent differences in the SEPs as a function of time caused by changes in the tactile stimulation location, classification results using the SEPs were generally insignificant. Table 1 shows the decoding performance using the temporal waveforms of SEPs. Here, the location of the tactile stimulation was determined by each temporal trace of a particular color (Figure 3). Classification was performed with the LDA classifier and 10-fold cross validation. In Table 1, each percentage was the highest correct rate achieved with the 63 electrode recordings. Because they were generally low, we did not continue using the SEPs in the rest of the study.

**Figure 4:**
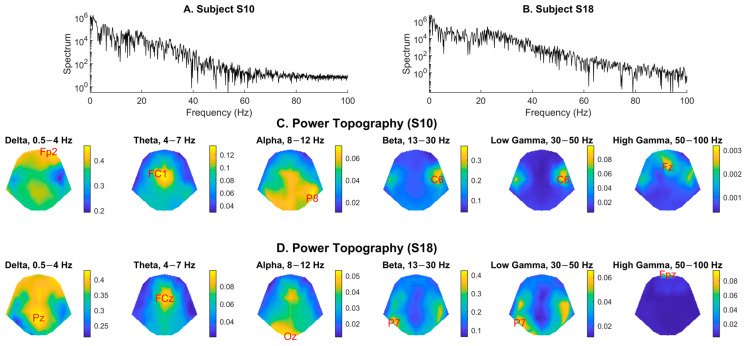
Power spectra (**A**,**B**) and topographies (**C**,**D**) for various EEG frequency bands from two representative subjects (S10, S18). In (**A**,**B**), the power spectra were derived with one full recording session obtained with Electrode Cz as examples. The tactile stimulation intensity was set low. In (**C**,**D**), the electrode name printed on each plot indicates the electrode showing the largest power.

Next, we mapped out the power distribution for each frequency band to see where most of that brainwave energy appeared. Figure 4A,B shows the power spectra over the first recording sessions (13.7 s) obtained with Electrode Cz for two representative subjects (S10 and S18) as examples. As always, EEG energy decreases with signal frequency [38,43].

Figure 4C,D shows the topography of EEG power. The tactile stimulation intensity was low. The power distributions generally agreed with previous studies [38,44,45]. Specifically, delta waves appeared mostly at the frontal (e.g., Fp2) and parietal lobes (e.g., Pz). The strongest theta wave was observed at the frontocentral locations (e.g., FC1, FCz). Alpha waves peaked around the occipital (e.g., Oz) or parietal lobes (e.g., P8). Beta and low gamma waves both featured the motor and somatosensory cortices (e.g., C6) and posterior-temporal (e.g., P7) lobes. High gamma waves mostly appeared at the frontal (e.g., Fpz) or frontocentral (e.g., Fz) locations. When the stimulation intensity was high, patterns were generally similar to the low-intensity plots for each subject. Note that a high power does not necessarily generate a high tactile classification result. For example, large alpha waves are typically observed at the occipital lobe [38], but they are more directly related to visual input, rather than tactile stimulation. Later, we will apply ANOVA analysis to examine the most useful electrodes for each frequency band related to the tactile-classification task.

### 3.2. Classification Using Single Frequency Bands with LDA

**Figure 5:**
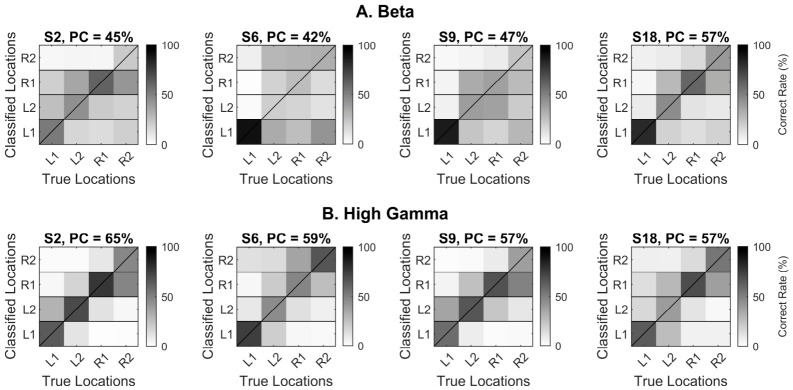
Confusion matrices for four representative subjects obtained with the low stimulation intensity for the beta (**A**) and high-gamma (**B**) bands. Results were obtained using the LDA classifier. PC, overall percent correct.

For each frequency band, the 63 power values obtained with 63 active EEG electrodes formed an input-feature vector, x (Equation (1)). The LDA algorithm combined with the 10-fold cross-validation evaluation method was then applied to classify the tactile locations. Figure 5 shows representative confusion matrices obtained with the beta (A) and high-gamma (B) bands for four human subjects, S2, S6, S9, and S18. The two frequencies were chosen for demonstrations because they yielded the highest overall performance. The tactile vibration intensity was low. Dark colors on the diagonals indicate correct decisions; dark colors off diagonal indicate incorrect decisions. The overall accuracy (i.e., percent correct) is the sum of the diagonal values normalized by the total number of incidences (i.e., 2000). It is also the γ in Equation (2).

A notable observation was that not all tactile locations were equally decoded with a given frequency band. For example, 3 out of the 4 subjects (S6, S9, and S18) here showed the highest accuracy for the upper-left arm location (Figure 4A, L1). This was not the case when the frequency band changed. Later, we will analyze the “preferred locations” for all conditions and subjects.

**Figure 6:**
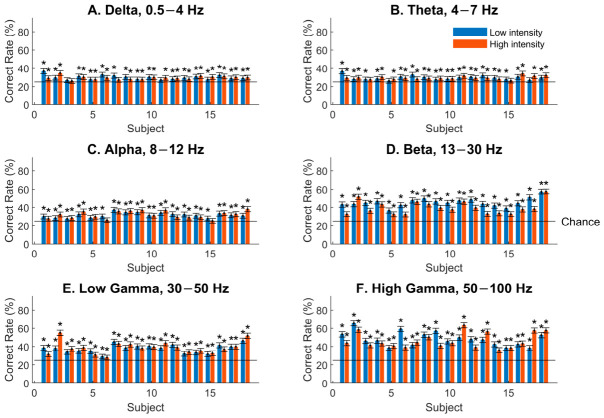
Tactile-location classification using power values of individual EEG frequency bands with the LDA classifier. Error bars are 99% confidence intervals. Asterisks indicate statistical significance (*p* < 0.01) using the confidence-interval test. Chance performance is 25% with four locations.

Figure 6 shows the overall classification performance derived from the confusion matrices for all the frequencies, stimulation intensities, and human subjects. Blue and orange bars are obtained with the low and high intensities, respectively. Error bars are 99% CIs derived using Equation (2). A statistical significance (marked with asterisks) was reached when the lower bound of the CI is above chance level (i.e., 25% for four locations). For the three lowest frequencies (A–C, delta, theta, and alpha), although most subjects showed significant performance, the detection was barely above the chance level.

The performance dramatically improved when the three higher-frequency bands were considered (Figure 6D,E). All subjects showed significant results for both the low (blue) and high (orange) intensities. Table 2 shows the average correct rates across all the subject for each frequency band. Beta and high gamma were the best two frequency bands. Note that although the classification accuracy obtained with the low stimulation intensity was often higher than with the high intensity (Table 2), this observation did not hold when the ANN classifier was applied.

We would like to point out that the vibration frequency of the tactors was set as 25 Hz, falling inside the Beta band. This could be a factor for why beta was the second-best frequency in terms of the decoding accuracy. We will discuss about an “entrainment effect” in Section 4.2 that may be related to this topic.

### 3.3. Classification Using Single Frequency Bands with ANN

**Figure 7:**
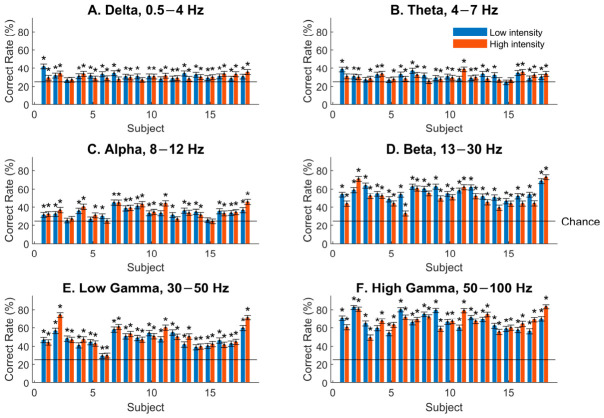
Tactile-location classification using power values of individual EEG frequency bands with the ANN classifier. Error bars are 99% confidence intervals. Asterisks indicate statistical significance (*p* < 0.01) using the confidence-interval test. Chance performance is 25% with four locations.

The above results were obtained with a linear LDA classifier. To examine whether some of the conclusions depended on the classification algorithm, we re-classified the data using an ANN with its structure laid out in Figure 2. Figure 7 shows the classification performance for individual frequency bands using the ANN. The general observation remained the same that the high-gamma band generated the best decoding results, ranging between 50% and 83% (Figure 7F).

However, it is no longer the case that the low-intensity results outperformed the high-intensity results, as previously observed using the LDA classifier. Table 3 shows the ANN results averaged over all the subjects. For the three highest frequency bands, the high-intensity accuracy values can be similar or even higher than the low-intensity results. In other words, the intensity effect was inconsistent and dependent on the classifier.

Unfortunately, the ANN cannot be used to generate the frequency-combined results below, because the combination would require 126 input-layer neurons for the ANN structure shown in Figure 2. We did not have sufficient data samples, even with 2000 trails, to train an ANN of that size. Therefore, we chose the LDA classifier in the frequency-combined test.

### 3.4. The “Best Electrodes”

**Figure 8:**
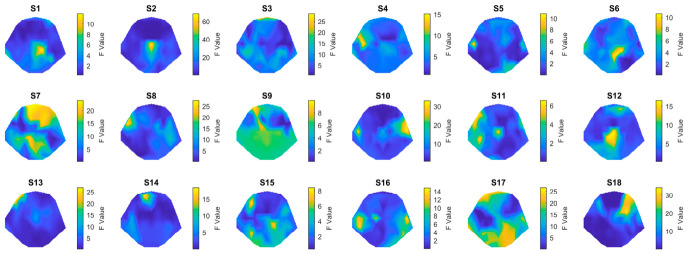
Topography of *F* values obtained with one-way ANOVA for the high-gamma power at the low-stimulation intensity. Warm colors represent higher sensitivity of the power values to the tactile locations.

The above decoding results were performed with all 63 electrodes forming a single input vector. To examine possible contributions of each electrode, a one-way ANOVA was applied to each electrode independently with the tactile location being the examined variable. A high *F* indicates a high ratio of between-group variance to within-group variance. Since the high-gamma band provided the highest accuracy and is the focus of this study, we plotted the *F* values obtained with the high gamma for all the subjects (Figure 8). In contrast to the gamma-power distribution (Figure 4, the rightmost column), the electrodes that were most sensitive to the tactile locations were not necessarily the electrodes showing the highest gamma power.

In addition, different subjects showed different distribution patterns of *F* (Figure 8). For this reason, when using the high gamma, the tactile information must be decoded by all the electrodes rather than with a few electrodes.

### 3.5. Combining Frequencies

**Figure 9:**
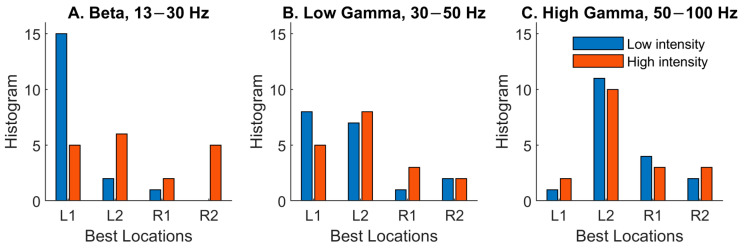
Histograms for the best decoded locations. Each bar indicates the number of human subjects that showed the best classified location using different frequency bands.

As shown in the confusion matrices (Figure 5), not all locations were equally decoded with the same EEG frequency and/or stimulation intensity. Figure 9 plots the histograms of the best decoded tactile locations for the three highest frequencies that showed decent performance. The histograms were created by counting the number of subjects that showed the best result for that location, derived from the corresponding confusion matrix. At each frequency (Figure 9A–C), the four locations were apparently not equally decoded.

To examine the possibility that different frequency bands may optimally encode different locations, we performed a correlation analysis to quantify how much redundant information existed across the three highest frequency bands. For each subject and each frequency band, the 63-feature input vector for both the LDA and ANN classifiers (Equation (1)) were averaged across 2000 trials. We then computed the correlation coefficient between each pair of the power vector, x, for the three highest frequency bands (Table 4).

The correlation values were highly significant between the beta and low-gamma bands (Table 4, marked by asterisks). In contrast, no correlation was found between the high-gamma and the other two frequency bands. That is, the high-gamma band did not seem to carry redundant information that was already contained in the lower-frequency bands. Therefore, we combined the beta ($\emptyset_{i}$) and the high-gamma bands (${\emptyset^{\prime }}_{i}$) to construct a new input vector of 126 features, which contained 63 power values of the beta and 63 of the high-gamma band.

(4) $$\hat{x}=[\emptyset_{1}\;\emptyset_{2}\;\cdots \;\emptyset_{63}\;{\emptyset^{\prime }}_{1}\;{\emptyset^{\prime }}_{2}\;\cdots \;{\emptyset^{\prime }}_{63}]$$

**Figure 10:**
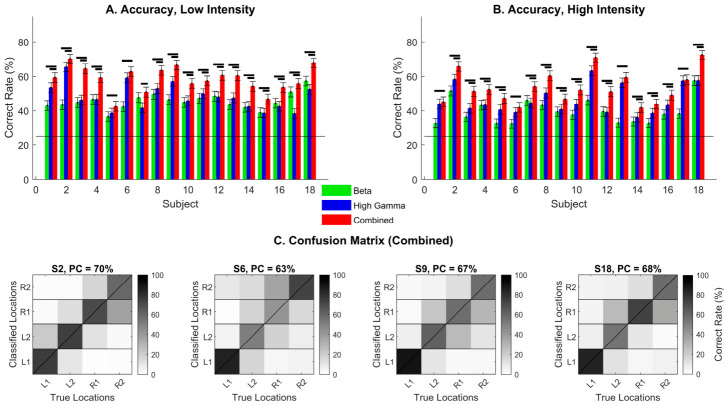
(**A**,**B**), comparisons of classification performance between the frequency-combined metric (red) and two individual frequencies using the LDA classifier. The green and blue bars are replotted from Figure 6D,F, the low intensity condition. Error bars, 99% confidence intervals. Horizontal lines, significant improvements using the *z* test (*p* < 0.01, with Bonferroni Correction). (**C**), confusion matrices obtained with combined frequencies for the four subjects in Figure 5.

Now, $\hat{x}$ served as the combined input to the LDA classifier. In Figure 10A,B, the red bars are the frequency-combined accuracies. The green and blue bars are replotted from Figure 6D,F, representing the beta and high-gamma performances. The short horizontal black lines mark significant increases (Equation (3), *z* test, *p* < 0.01, with Bonferroni corrections) by combining the two frequency bands. All subjects showed improved performances. The highest individual accuracy was 71% and 72% for the low and high intensities, respectively.

Figure 10C shows the new confusion matrices obtained with combined frequencies. When comparing with the single-frequency results (Figure 5), we can see that the diagonal values (i.e., correct classifications, Figure 10C) may resemble the best performance in the individual-frequency results, such as Location L1 (Figure 5). The combined performance in Figure 10C can also be notably improved than the individual performance, such as Location R2 (Figure 5).

Table 5 summarizes the average performance across subjects. Typically, higher EEG frequency components indicate activity from smaller local groups. The fact that the combined-frequency performance is notably improved compared with either of the individual frequency band hints that locational information across the two arms is probably not encoded by a single group of neurons. The reason for choosing these two frequencies was because (1) they generally showed higher performance than the low-gamma band, and (2) the power derived from the high-gamma band was uncorrelated to the lower-frequency bands. Indeed, when we tried combining the low gamma with either of the other two, the combined performance was not as good as what was obtained in Table 5.

Note that even the combined and improved performance using the LDA classifier (Table 5) was lower than the high-gamma performance obtained with the ANN (Table 3). Unfortunately, we were unable to train an ANN to accommodate the 126-feature input vector, $\hat{x}$ (Equation (4)). That would require 126 neurons in the input layer and more neurons in the hidden layer (Figure 2). With the amount of available data samples (i.e., 2000), an ANN of that size cannot be sufficiently trained.

### 3.6. Stimulation Intensity and Gender

**Figure 11:**
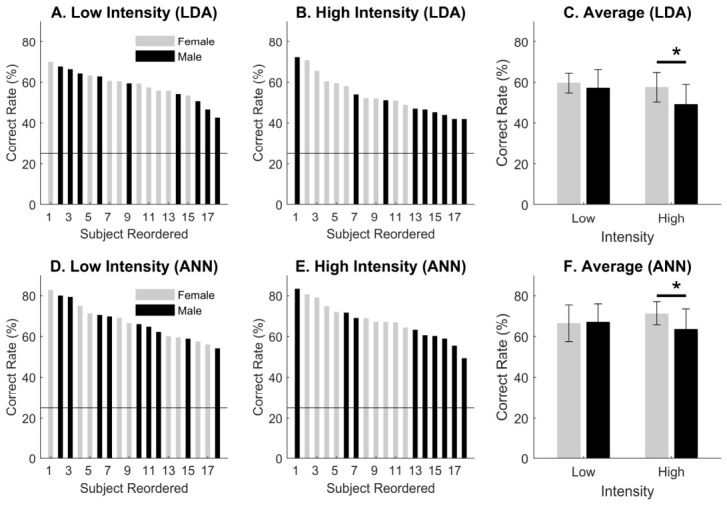
Effect of gender on the tactile-location decoding performance using the LDA (**A**–**C**) and ANN (**D**–**F**) classifiers. (**A**,**B**), the same combined performance using the LDA classifier (Figure 10A,B, red) is sorted from high to low. Female and male subjects are represented with gray and black bars, respectively. (**C**), performance using the LDA classifier averaged over subjects for each gender. (**D**,**E**), the same high-gamma performance using the ANN classifier (Figure 7F) is sorted from high to low. (**F**), performance using the ANN classifier averaged over subjects for each gender. Error bars are standard deviations over 9 subjects for each gender. In (**D**,**F**), the average female performance was significantly higher than the average male performance under the high-stimulation condition, marked by the asterisks (*z* text, *p* < 0.01).

In our previous EEG study of decoding tactile locations on the legs and buttocks [34], female subjects generally showed higher decoding accuracy than male subjects. Therefore, we also examined the gender effect for the present study. Figure 11A–C rearranged the frequency-combined performance using the LDA classifier from the highest to the lowest for both stimulation intensities. Dark and gray bars were correct rates achieved by male and female subjects, respectively (Figure 11, A and B). For the high stimulation intensity (Figure 11B), there is a clear tendency for female subjects to yield higher accuracies. The average performance within each gender is plotted in Figure 11C. Female subjects showed higher but insignificant performance than male subjects with the low-intensity stimulation (Figure 11C, left). The decoding performance of female subjects was significantly higher (*z* test, *p* < 0.01) for the high-intensity stimulation (Figure 11C, right).

Figure 11D,E was obtained with the ANN classifier using the high-gamma with resorted performance. The sorting patterns varied slightly from Figure 11A,B but the general conclusion remained the same. A significantly higher average performance was observed with female subjects only under the high-intensity condition (F).

## 4. Discussion

### 4.1. EEG and Tactile Remapping

EEG-based decoding approaches have been used previously to investigate somatosensory representations and tactile perception, including applications involving vibrotactile stimulation and brain–computer interfaces [46,47,48]. Advances in machine-learning algorithms and high-density EEG recordings have further improved the feasibility of decoding tactile information from non-invasive neural signals. In the tactile-EEG study by Yakovlev, et al. [49], sensorimotor rhythms (i.e., mu and beta waves) can be reduced by actual and imagined tactile stimuli.

Regarding the stimulation protocols and analysis approaches, tactile-EEG studies can be generally classified into two categories. The present study and a few other studies, which may or may not relate to tactile locations, used vibrotactile stimulation to evoke steady-steady responses [24,25,34,48]. Given the nature of the long-lasting stimulations, the frequency power or spectral analysis of various frequency bands is frequently used as the EEG signal feature for decoding the tactile input. However, temporal SEP waveforms have also been used in the analysis of vibratory stimulations, such as attentional selectivity in touch [50].

The second category is the temporal approach that focuses on various peaks and troughs in the SEP waveforms, specifically the P300 [46,47]. In those studies, the tactile stimulation is typically very brief, generating evoked temporal responses that are precisely timed. Of particular interest are the tactile remapping studies that combine skin stimulations, EEG, and body postures [1,22,23]. Those studies did not provide classification algorithms that can actually decode the tactile locations. The dominant view is that extrinsic encoding strategies or exteroceptive perception underlie tactile remapping [1,8,14]. For example, a tactile input received on the right hand will elicit different brain signals when the right hand is placed on the left verses the right side of the body. Other researchers believe that intrinsic encoding or interoceptive perception dominants tactile remapping [22].

Regardless of the hypotheses, those studies commonly analyze the distributions of somatosensory-evoked peaks, negative or positive, during various time frames after the tactile input. The evoked responses always propagate through the entire head, not just over the parietal lobe where the somatosensory cortex is located [1,23]. Therefore, it is not surprising that the best decoding strategy in the present study is to use the signal power of all 63 electrode channels as the input feature, rather than choosing only a few electrodes. Even though the ANOVA topography shows that certain electrodes provide better locational results than other electrodes (Figure 6), reducing the number of electrodes always yielded worse results.

In general, the perception of tactile stimulation can be a dynamic process, as multiple studies have demonstrated how body posture, attention, and multisensory interactions influence tactile-localization performance [14,51,52,53]. For example, attention can modulate the gating of primary somatosensory oscillations [54]. In that study, directing attention to the somatosensory domain enhanced gating of the early theta response and reduced gating of the later alpha and beta responses. They did not examine the gamma band. In the present study, we did not implement any mechanism to control the subjects’ attention, which could be a factor for the observed inter-subject performance diversities.

### 4.2. Legs vs. Arms

**Figure 12:**
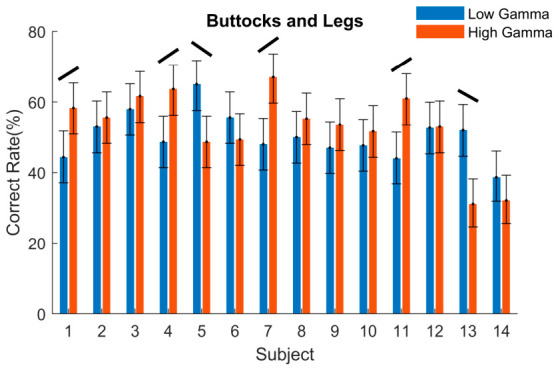
Comparing the classification performance for tactile stimulation on the buttocks and legs (data from [34] was reanalyzed) between the low and high gamma using the ANN. Horizontal lines tilted upwards indicate significantly higher (*z* text, *p* < 0.01) performance with the high gamma. Horizontal bars tilted downwards indicate significantly lower (*z* text, *p* < 0.01) performance with the high gamma. Error bars are 99% confidence intervals.

In our previous study [34], we attached four vibrotactile stimulators to the legs and buttocks along the sitting interface. We found that the signal power of beta and low-gamma bands generated the best decoding accuracy. After the human subjects sat in a chair for one hour or longer, degraded locational sensitivity was observed with a foam cushion more than with an air-cell cushion. The latter was supposed to alleviate stress over prolonged sitting. At that time, we did not analyze the high-gamma band. To provide a fair comparison for the present study, we revisited the recordings with leg stimulations and extracted the high-gamma signals. Figure 12 shows the classification results using the low (blue) and high (orange) gamma bands. Only recordings made at the beginning of the sitting period are analyzed here. Out of the 14 human subjects, 10 showed increased performance with the high-gamma band, but only four were statistically significant (*z* test, *p* < 0.01). One possibility is that fewer trials (i.e., 500) were obtained in the leg study compared with the present arm study (i.e., 2000), making it more difficult to reach the significant value. This could be a limitation of the previous study.

Nevertheless, for both arm and legs, our results showed that the gamma band, especially the high-gamma band, provides the best location information with tactile stimulations. Here, we must point out a MEG study that applied 20-Hz vibrational stimulations to their subjects’ fingertips [55]. They found entrainment of oscillations (i.e., enhanced amplitude) in the beta band (13–30 Hz), as expected. Interestingly, they also found larger amplitude in the gamma band with repetitive stimulations, even though the stimulation frequency (i.e., 20 Hz) was far below the gamma band. In the present study, because each subject completed 50 trials for every location in each recording session with a vibration frequency of 25 Hz, it is possible that both beta and gamma bands had gone through similar entrainment process.

It should be noted that the gamma frequency may contain signals from electromyogram (EMG). Although certain EEG electrodes may pick up EMG signals originating from the head, those signals did not contain tactile-location information for the arms and thus could not contribute to the signal classification shown above. However, the EEG electrodes may also pick up EMG signals coming from the arm that were related to the tactile locations. That said, since we used the common average montage, those EMG signals should also exist in the average signal that was subtracted out from all the active electrodes. Thus, we expect the contribution of arm-EMG signals to be minimized in our signal classification. Nevertheless, it remains a limitation of the present study, especially considering the gamma frequency band.

### 4.3. Combining EEG Frequencies

Examining the confusion matrices for individual-location performance (Figure 4), we found that different frequency bands encoded the four locations with different preferences (Figure 7). In addition, the correlation analysis showed that the power information contained in the high-gamma band was non-redundant with respect to the information contained in the lower frequencies. We suspect that even though the high-gamma band provided the best performance overall, certain tactile locations (e.g., L1 with the low stimulation intensity) may be best encoded by other frequency bands. This finding led to our attempt to combine more than one frequency into a super input vector with 126 features. Indeed, the decoding performance was significantly improved for every subject when beta was combined with high-gamma bands (Figure 10A,B). We have also tried combining beta and high gamma or low and high gamma but failed to match the above improvement.

The confusion matrices when frequencies were combined (Figure 10C) indicate that this improvement was achieved by further enhancing the “best decoded location” from each individual frequency band, not just a simple summation. For example, L1 was the best location for Subject S6 using the beta-band power individually (82%; Figure 5A, second left) and R1 was the best location using the high-gamma band (69%; Figure 5B, second left). In the frequency-combined confusion matrix, the accuracies for the two locations were increased to 90% and 74%, respectively (Figure 10C, rightmost). The finding suggests that the two frequencies, namely beta and high gamma, work together to create a better decision variable for tactile locations.

In the EEG-arm study by Wang, et al. [25] and the EEG-finger study by He and Contreras-Vidal [24], they were both capable of achieving decoding accuracies above 95%. The first study applied wavelet-packet Entropy to their bandpass filtered signals, followed by a support-vector-machine classifier. The second study constructed a 704-dimention feature vector, applied the LDA for dimension reduction, and used a Gaussian Mixture Model for classification. The advantage of our algorithm lies in its simplicity—we simply computed the signal power and used it as the input to the LDA. However, we need to caution that the four tactile locations were spaced quite apart from one another in this study (i.e., upper-left arm, lower-left arm, upper-right arm, lower-right arm; Figure 1). The tactile-attention EEG study by Eimer and Forster [50] showed that, when fingers of the same hand are stimulated and the person’s attention is altered, it matters whether the fingers are adjacent or non-adjacent. Therefore, whether the findings in the present study, especially the best-location and frequency-combination results, can generalize to tactile stimulations that are closely positioned needs more investigations.

### 4.4. The Gender Effect

Studies have shown that young females generally have a higher density of mechanoreceptors [56], which leads to better tactile perception, discriminability and acuity [57,58], etc. In our previous leg EEG study, we found that the decoding performance obtained from female subjects not only had higher accuracy but also sustained better after prolonged sitting than male subjects [34].

In the present study, gender played a significant effect under the high-intensity condition (Figure 11) using both the LDA and ANN classifiers. We should caution that, since the sample size was limited (i.e., 18 subjects in total), the observation may not hold for underage or older people, given that we only recorded EEG from the age group of 19–26.

### 4.5. Summary and Conclusions

The present study explored a simple decoding algorithm that uses multi-channel EEG power to decode the location of tactile stimulations on the two arms. The basic finding agreed with our previous study on the legs in that beta and gamma frequencies provide the most useful information for the classification of locations. An interesting finding is that combining the beta and high-gamma bands significantly improved the prediction accuracy because the high-gamma band carried power information that was uncorrelated to the lower frequencies. This finding may indicate that different locations can be optimally decoded with different frequency bands, but more experiments need to be performed in the future to directly examine this possibility. Our findings agree with those previous studies that have demonstrated the importance of the gamma frequency during tactile perception.

## Figures and Tables

**Table 1:** The best classification accuracy using the SEP temporal waveforms over all 63 electrode recordings for each subject. The stimulation intensity was low.

Subject	S1	S2	S3	S4	S5	S6	S7	S8	S9
Correct Rate	33%	29%	29%	29%	28%	31%	34%	31%	29%
Subject	S10	S11	S12	S13	S14	S15	S16	S17	S18
Correct Rate	31%	32%	29%	28%	31%	28%	27%	30%	28%

**Table 2:** Average performance (±standard deviation) across subjects for individual frequency bands at low or high stimulation intensities. Results were obtained with the LDA classifier and 10-fold cross validation.

Classification Performance Using LDA and Single Frequencies						
	Delta	Theta	Alpha	Beta	Low Gamma	High Gamma
Low Intensity	30% (±2%)	30% (±2%)	31% (±3%)	46% (±4%)	38% (±5%)	49% (±8%)
High Intensity	29% (±2%)	29% (±2%)	32% (±4%)	39% (±8%)	38% (±7%)	46% (±8%)

**Table 3:** Average performance (±standard deviation) across subjects for individual frequency bands derived with the ANN and 10-fold cross validation.

Classification Performance Using ANN and Single Frequencies						
	Delta	Theta	Alpha	Beta	Low Gamma	High Gamma
Low Intensity	31% (±3%)	31% (±4%)	34% (±5%)	57% (±6%)	47% (±8%)	67% (±9%)
High Intensity	30% (±3%)	30% (±3%)	35% (±7%)	51% (±10%)	50% (±11%)	68% (±9%)

**Table 4:** Pearson’s Correlation Coefficients of the power vector, *x*, between pairs frequency bands. The values are averages over all the subjects (±standard deviation). * Statistically significant (*df* = 61, *p* < 0.01). Bonferroni Corrections were applied for multiple comparisons.

Stimulation Intensity	Beta vs. Low Gamma	Beta vs. High Gamma	Low vs. High Gamma
Low	0.90 * (±0.17)	0.11 (±0.33)	0.09 (±0.30)
High	0.93 * (±0.07)	0.03 (±0.33)	0.06 (±0.31)

**Table 5:** Average performance (±standard deviation) over all subjects for beta, high gamma, and combined frequencies using the LDA classifier.

	Beta	High Gamma	Combined
Low Intensity	45% (±5%)	48% (±8%)	58% (±7%)
High Intensity	40% (±7%)	47% (±8%)	54% (±9%)

## Data Availability

MATLAB codes and EEG data can be found at the following online data repository, https://doi.org/10.7910/DVN/NELUGC.

## Abbreviations

The following abbreviations are used in this manuscript:

ANN	Artificial neural network
CI	Confidence interval
ECoG	Electrocorticography
EEG	Electroencephalography
EMG	Electromyogram
fMRI	Functional magnetic resonance imaging
ICA	Independent Component Analysis
LDA	Linear discriminant analysis
MEG	Magnetoencephalography
SEP	Somatosensory-evoked potential
